# Molecular Structure and Decomposition Kinetics of Kaolinite/Alkylamine Intercalation Compounds

**DOI:** 10.3389/fchem.2018.00310

**Published:** 2018-07-27

**Authors:** Yi Zhou, Qinghe Liu, Peijie Xu, Hongfei Cheng, Qinfu Liu

**Affiliations:** ^1^School of Geoscience and Surveying Engineering, China University of Mining & Technology, Beijing, China; ^2^Department of Chemical and Biomolecular Engineering and Polymer Program, Institute of Materials Science, University of Connecticut, Storrs, CT, United States; ^3^School of Environmental Science and Engineering, Chang'an University, Xi'an, China

**Keywords:** kaolinite, alkylamine, intercalation, structural model, decomposition

## Abstract

Although the development of clay/polymer nanocomposites and their applications have attracted much attention in recent years, a thorough understanding of the structure and the decomposition mechanism of clay/polymer nanocomposites is still lacking. In this research, the intercalation of kaolinite (Kaol) with different alkylamines were investigated by X-ray diffracion (XRD), Fourier-transform infrared spectroscopy (FTIR), and thermogravimetry and differential scanning calorimetry (TG-DSC). The results showed that the intercalation of Kaol/methanol compound with hexylamine (HA), dodecylamine (DA), and octadecylamine (OA) led to the expansion of the interlayer distance and resulted in the dominant basal diffraction at 2.86, 4.08, and 5.66 nm. The alky chains of HA, DA, and OA are tilted toward the Kaol surface in bilayer with an inclination angle of ~40°. The most probable mechanism function, activation energy *E*, and pre-exponential factor *A* were obtained by mutual authentication using KAS and Ozawa methods, itrative and Satava integral method. The average activation energy *E* of the three intercalation compounds are 104.44, 130.80, and 154.59 kJ mol^−1^, respectively. It shows a positive correlation with the alkyl chain length. The pre-exponential factor *A* was estimated to be 1.09 × 10^15^, 1.15 × 10^8^, and 4.17 × 10^21^ s^−1^, respectively. The optimized mechanism function for the decomposition of alkylamine is *G(*α*)* = [(1-α) ^−1/3^−1]^2^.

## Introduction

Design and fabrication of clay/polymer nanocomposites have attracted high interest from both the scientific and engineering communities (Cheng et al., 2017). A wide variety of clay/polymer nanocomposites have been developed and are being used in widespread application (Gogoi and Raidongia, 2017). Pre-intercalating clays followed by the incorporation of polymers into clay minerals layer is a widely adopted approach for the preparation of clay/polymer nanocomposites (Li et al., 2009; Zare et al., 2017). While being intercalated within inorganic clay layers, organic polymer chains naturally reduce their structural mobility and some of them assume a highly organized conformation within the layered structure.

In nature, kaolinite (Kaol) is a type of clay mineral with a 1:1 dioctahedral aluminum silicate layered structure (Brindley and Robinson, 1945). The layers in Kaol are held together by hydrogen bonds, dipole-dipole interactions, and van der Waals forces (Brindley et al., 1967). However, only a limited number of highly polar organic species including urea (Makó et al., 2009), dimethyl sulfoxide (Costanzo and Giesse, 1986), formamide (Frost et al., 2000a), hydrazine (Cruz and Franco, 2000), and potassium acetate (Frost et al., 2000b) were successfully intercalated into the gallery of Kaol. The intercalation of small molecules into Kaol layers causes an increase in the basal spacing, and can be used as a preliminary expansion step for subsequent insertion of large-sized, non-reactive species by the displacement of the pre-intercalated small molecules (Cheng et al., 2015). For example, based on the previous reports, a Kaol/methanol intercalation compound can be an effective intermediate for further intercalation reaction with ethylene glycol (Hirsemann et al., 2011), hexylamine (Matusik et al., 2012), n-alkylamines (Gardolinski and Lagaly, 2005), and quaternary ammonium salts (Cheng et al., 2016). In addition, the majority of guest molecules are difficult to be inserted into the interlayer space, one of the reason is the strong hydrogen bonds, another is that there is no exchangeable ions in the Kaol structure.

Although clay/polymer nanocomposites have been well developed in recent years, to synthesize new clay/organic nanocomposites for potential application still presents a big challenge (Kotal and Bhowmick, 2015). In order to settle this problem, it is really an urgent matter to figure out the nano-scale process of kaolinite intercalation and make a thorough inquiry to the mechanism of decomposition with modern technology and analytical tools. Thermal analysis and kinetic calculation of clay/polymer intercalation compounds can help characterize the decomposition processes and provide scientific basis for control over intercalation reactions (Zhang et al., 2015). The kinetic parameters of the decomposition reaction kinetics process, such as the activation energy, pre-exponential, reaction orders, and rate constant were assessed with the data from clay/polymer thermograms. In this study, the intercalation of Kaol with various alkylamines was investigated. Additionally, the intercalation process and the decomposition mechanism was systematically investigated.

## Experimental

### Materials

Kaolin used in this study was exploited from Zhangjiakou, China. The main mineral composition is a well ordered kaolinite (95% in mass). It was ground to pass a 325-mesh sieve (particles that measure < 44 μm) before intercalation. Alkylamine hexylamine (HA, 99%), dodecylamine (DA, Chemically Pure), and octadecylamine (OA, Chemically Pure) were purchased from Nanjing Shuguang Chemical Company, China. Dimethyl sulfoxide (DMSO, Analytical Reagent), methanol (MeOH, Analytical Reagent), and toluene (99%) were received from Xilong Chemical Company, China, and used as received without further purification. The chemical formula and structural formula of HA, DA, and OA are shown in Table 1.

**Table 1:** Chemical and structural formula of HA, DA, and OA.

**Chemicals**	**Chemical formula**	**Structural formula**
Hexylamine (HA)	C_6_H_15_N	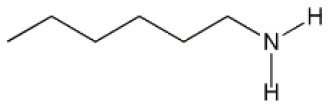
Dodecylamine (DA)	C_12_H_27_N	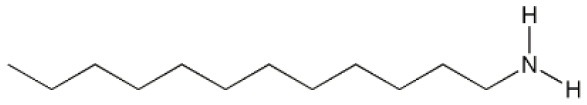
Octadecylamine (OA)	C_18_H_39_N	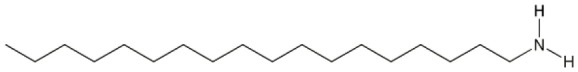

### Synthesis of intercalation compounds

First, Kaol/DMSO intercalation compound was prepared by dispersing 20.0 g Kaol into a mixture of 36.0 g DMSO and 4.0 g water. The mixture was stirred in a water-bath for 2 h at 95°C, and then the suspension was separated by centrifugation with ethanol. Second, the Kaol/DMSO intercalation compound was used as a precursor for further reaction with MeOH. MeOH was added to the pre-intercalated Kaol and the reaction mixture was stirred for 10 days, with MeOH being replaced each day with a same amount of fresh MeOH. The precipitate in the mixture was separated by centrifugation and dried in an oven at 60°C for 12 h to obtain Kaol/MeOH intercalation compounds. Finally, 2 g Kaol/MeOH intercalation compound was mixed with 30.0 mL HA, DA, or OA methanol solutions (1 mol/L) by stirring at ambient temperature, respectively. The dispersions were centrifuged after reaction for 24 h. The sediments were washed three times with toluene to remove the excessive HA, DA, or OA. The samples were dried at room temperature for 12 h and ground into powders with an agate mortar (Komori et al., 1999). The resulting compounds are labeled as Kaolpi/HA (pi as pre-intercalated), Kaolpi/DA, and Kaolpi/OA.

### Characterization

The XRD patterns were recorded on a Rigaku D/max 2500PC X-ray diffractometer with Cu Kα (λ = 1.54178 Å) radiation operating at 40 kV and 150 mA. The randomly oriented specimens samples were scanned in the 2θ range between 1 and 20° at a speed of 2° min^−1^. A Thermo Fisher Nicolet 6700 spectrophotometer were used to recorded the FTIR spectra within the range of 4,000~400 cm^−1^. The TG-DSC analyses were performed with a Mettler-Toledo TG-DSC I/1600 HT simultaneous thermal analyzer under nitrogen atmosphere. Twenty milligrams of sample was placed in an alumina crucible and heated from 30 to 1,100°C with a series of heating rates at 4, 6, 8, 10°C min^−1^.

## Results and discussion

### XRD characterization

**Figure 1:**
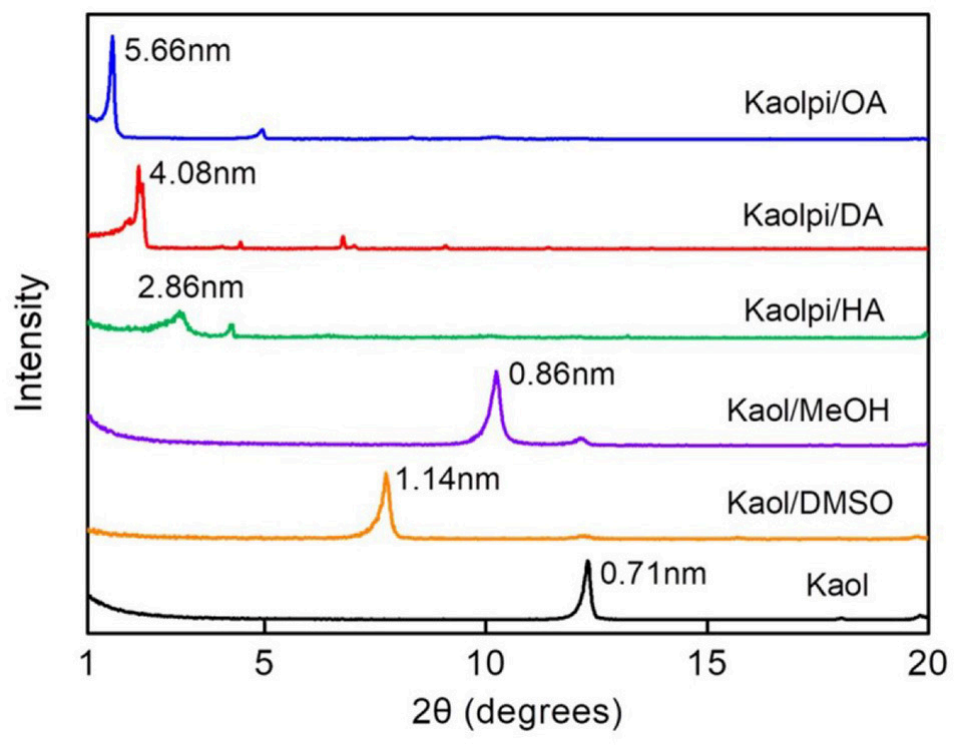
XRD patterns of Kaol, Kaol/DMSO, Kaol/MeOH, Kaolpi/HA, Kaolpi/DA, and Kaolpi/OA.

The XRD patterns of the pristine Kaol and its intercalation compounds are shown in Figure 1. The pattern of the pristine Kaol displays a well-ordered layered structure with a basal spacing [*d*_(001)_] of 0.71 nm. This value matches well with the standard ICDD reference pattern 14-0164 [kaolinite, Al_2_Si_2_O_5_(OH)_4_]. Upon being treated with DMSO, a new basal reflection peak appeared at 1.14 nm, indicating that DMSO was successfully inserted into Kaol interlayers. After the Kaol/DMSO intercalation compound was treated with MeOH, the 1.14 nm (001) reflection characteristic of Kaol/DMSO intercalation compound was shifted to 0.86 nm. It was reported by Matusik et al. (2012) that Kaol/DMSO/MeOH compound was dried at 110°C, the reflection with *d* = 1.12 nm disappeared and a broad reflection with maximum at 0.95 nm was observed. Based on the chemical formula Al_2_Si_2_O_5_(OH)_3.20_(OCH_3_)_0.80_ of Kaol/DMSO intercalation compound which has been calculated according to the basis of CHNS analysis, it showed that about 1/3 of the inner surface OH groups were replaced by methoxy groups, and this observation also suggests the formation of a Kaol/MeOH intercalation compound (Tunney and Detellier, 1996; Cheng et al., 2015). The XRD patterns also show that the Kaol/MeOH compounds intercalated with HA, DA, and OA expands the structure along the *c*-axis, leading to a large interlayer distance of 2.86, 4.08, and 5.66 nm, respectively. The lengths of HA, DA, and OA molecular chains are 1.56, 2.51, and 3.65 nm, respectively (McNulty et al., 2011, 2014). Overall, the interlayer distance of the Kaol/alkylamine intercalation compounds increased with the alkyl chain length of the alkylamines, but the alkylamine molecule is not a single layer or bilayer structure which is perpendicular to the Kaol surface.

### FTIR spectra

**Figure 2:**
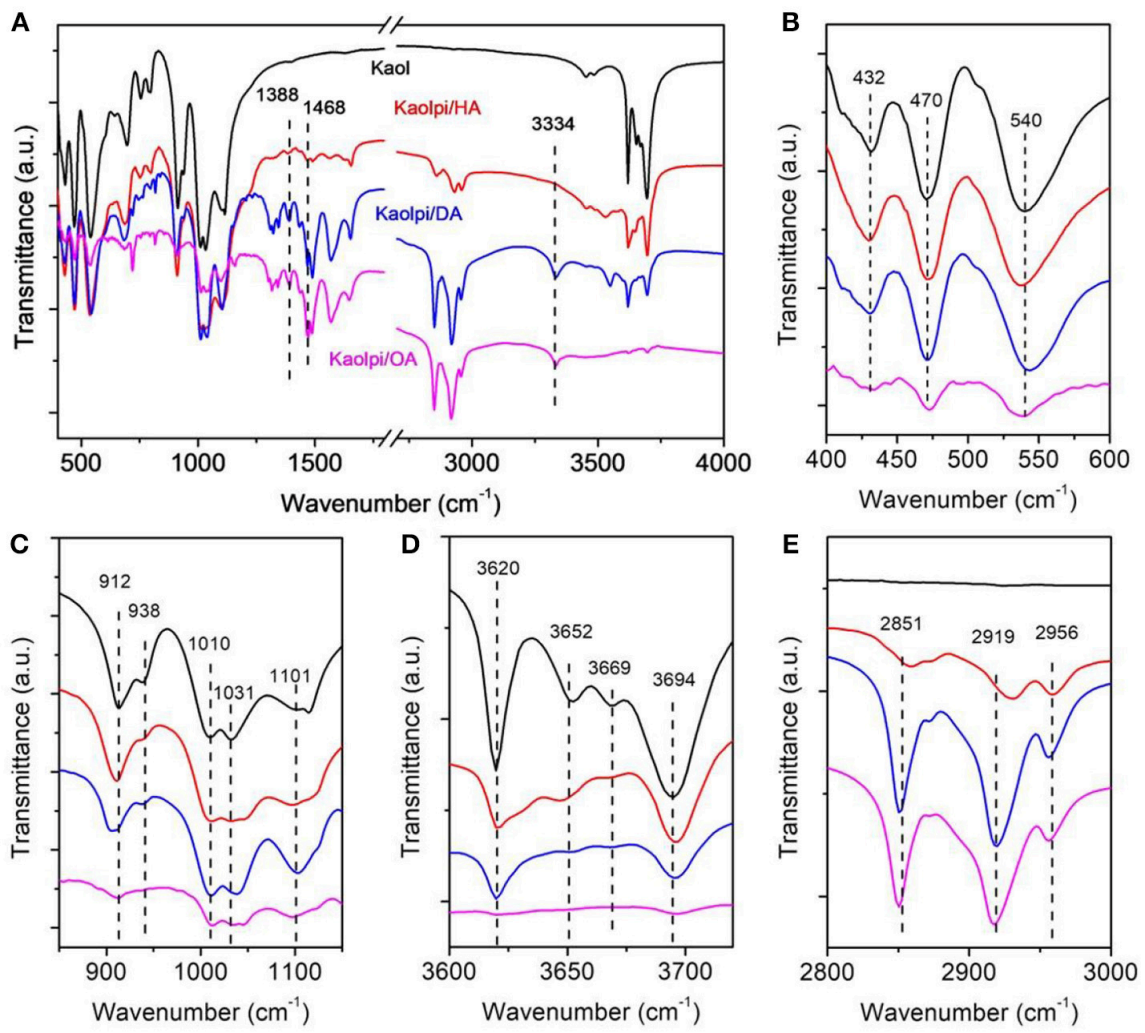
Infrared spectra of Kaol, Kaolpi/HA, Kaolpi/DA, Kaolpi/OA.

FTIR spectroscopy has been widely used in the characterization of intercalation compounds (Ledoux and White, 1964; Frost et al., 2000c; Cheng et al., 2012). The FTIR spectra of the original Kaol, Kaolpi/HA, Kaolpi/DA, and Kaolpi/OA intercalation compounds are shown in Figure 2. The characteristic bands at 432, 470, and 541 cm^−1^ (Figure 2B) belong to the deformation mode of Si-O, Si-O-Si, and Al-O-Si. The other bands at 1,009, 1,031, and 1,114 cm^−1^ (Figure 2C) are assigned to the stretching vibrations of Si-O-Si in the layer of Kaol. Comparing Kaol and its intercalation compounds spectra, the position of the bands have no apparent variation, which indicates that this stretching vibrations bands are not affected by the intercalated molecules.

The bands at 912 and 938 cm^−1^ (Figure 2C) belong to the OH deformation of inner hydroxyl groups and inner-surface hydroxyl groups. There are four obvious hydroxyl stretching bands at 3,694, 3,669, 3,652, and 3,620 cm^−1^ (Figure 2D) in the spectrum of the original Kaol, the bands at 3,694, 3,669, and 3,652 cm^−1^ are caused by the inner-surface hydroxyl groups while the one at 3,620 cm^−1^ results from the inner hydroxyl groups (Farmer, 1964; Ledoux and White, 1964; Giese and Datta, 1973). After the intercalation of alkylamine molecules, some bands were weakened due to the change of the interactions with neighboring atoms. The shift to 3,697 cm^−1^ and the decrease of the intensity of the band at 3,694 cm^−1^ indicates that numerous hydroxyl groups in Kaol participated and formed hydrogen bonds with the amine groups in the alkylamine molecules (Caglar, 2012; Caglar et al., 2013; Zhang et al., 2015).

The presence of interlayer alkylamine molecules was detected on the FTIR spectrum by the new peaks, which are shown in Figures 2A,E. For the intercalation compounds, some obvious characteristic peaks such as the band at 1,388 and 1,468 cm^−1^ belong to the C-H bending mode of -CH_3_ and -CH_2_, respectively. A new vibration band at 3,334 cm^−1^ was observed which is due to the stretching mode of the N-H stretching vibration (Griffiths and De Haseth, 2007; Zhang et al., 2018). The new vibration band at 2,956 cm^−1^ was observed and usually assigned to the C-H asymmetric stretching band of the terminal methyl groups (Cheng et al., 2016). The two vibration bands at 2,919 and 2,851 cm^−1^ are usually assigned to the symmetric and asymmetric stretching vibrations of the -(CH_2_)_n_- (Venkataraman and Vasudevan, 2001). The intensity of the bands increases gradually which means more alkylamine carbon atoms has been inserted into Kaol interlayers. Furthermore, the volatility of alkylamines will decrease with the increase of carbon chain, so the stability of the corresponding Kaol intercalation compounds will gradually increase with larger alkylamine molecular mass (Wang et al., 2010).

### Structural model of Kaol/alkylamine intercalation compounds

The arrangement of intercalated molecules is the foundation for experimental analysis of the Kaol/alkylamine intercalation compound structure. According to Lagaly (1981), the montmorillonite-quaternary ammonium salt intercalation compounds structural models can be divided into three categories: (a) monolayers: short chain alkylamine ions; (b) bilayers: long-chain quaternary ammonium ions; (c) three-layers: kinked alkyl chains in highly charged clay minerals. Beneke and Lagaly (1982) further proposed different structure of the intercalated alkylamine molecules based on the XRD, which are summarized as follows: (a) the alky chains in the interlayer space are tilted to the (001) crystal plane; (b) the alky chains are arranged in bilayers. Brindley and Moll (1965) described that the intercalated molecules are tilted to the silicate sheets in a single layer, whereas the angle is about 65° to (001) crystal plane. If the interacting molecules are uniformly attached to all silicate surfaces by the active hydroxyl groups, an “end to end” organization in pairs with some longitudinal displacement is suggested. The incline angle is in accord with the possible close packing manner between the silicate oxygen surfaces and the terminal hydroxyl groups and the degree of close packing of chain molecules among themselves.

**Figure 3:**
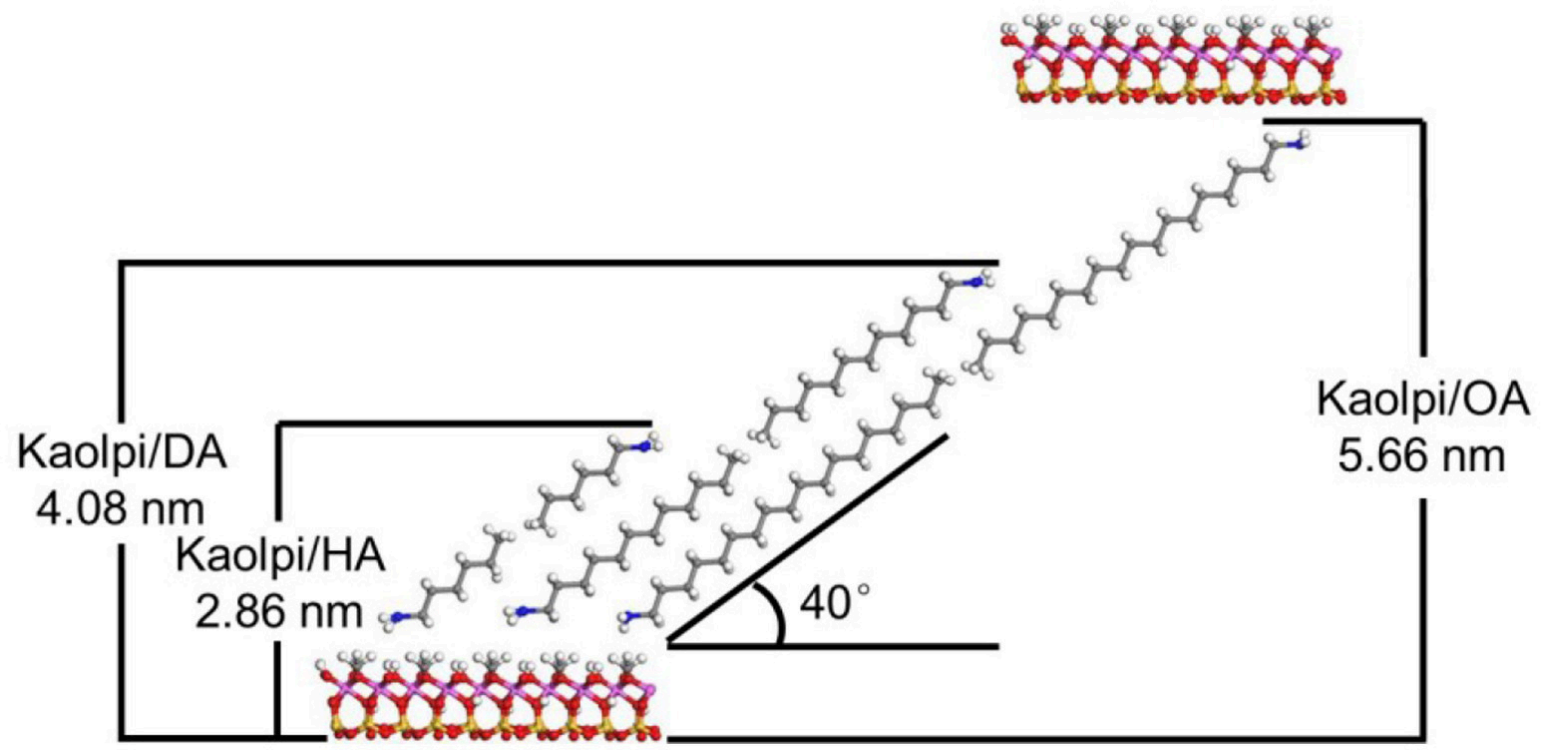
The most possible structural model for the kaolinite/alkylamine intercalation compounds.

The alkyl molecules are hard to intercalate directly the original Kaol layers since it lacks exchangeable ions in interlayer space of Kaol. Valid precursors are essential when the alkylamine molecules insert into the interlayer of Kaol. It was reported that the alkyl trimethyl ammonium chloride chain forms a tilted bilayer in the interlayer of Kaol and the angle of tilt is ~36° after calculation (Kuroda et al., 2011). Yuan et al. (2013) pointed out that the structural arrangement of the quaternary ammonium salt molecules in the interlayer of Kaol tilted bilayer and the angle of tilt is ~38.4°. Gardolinski and Lagaly (2005) proposed that the alkyl chains in the interlayers are fully stretched and oriented perpendicular to the Kaol surface by bilayer. Therefore, from analyzing the length of HA, DA, and OA molecules chain, the conclusion can be drawn that the basal spacing after intercalation is not sufficient to allow the alky chain entering by vertical and bilayer manner. It can be seen by further analyzing the XRD results that the alkyl chains of HA, DA, and OA are tilted to the Kaol surface in bilayer and the incline angle of the alky chains are about 39.9, 39.9, 41.1°, respectively. In addition, due to the hydroxyl bond between inner-surface hydroxyl in Kaol and amine groups in alkylamine molecules, the most possible structural model for the Kaol/alkylamine intercalation compounds is shown in Figure 3.

### Thermal analysis

**Figure 4:**
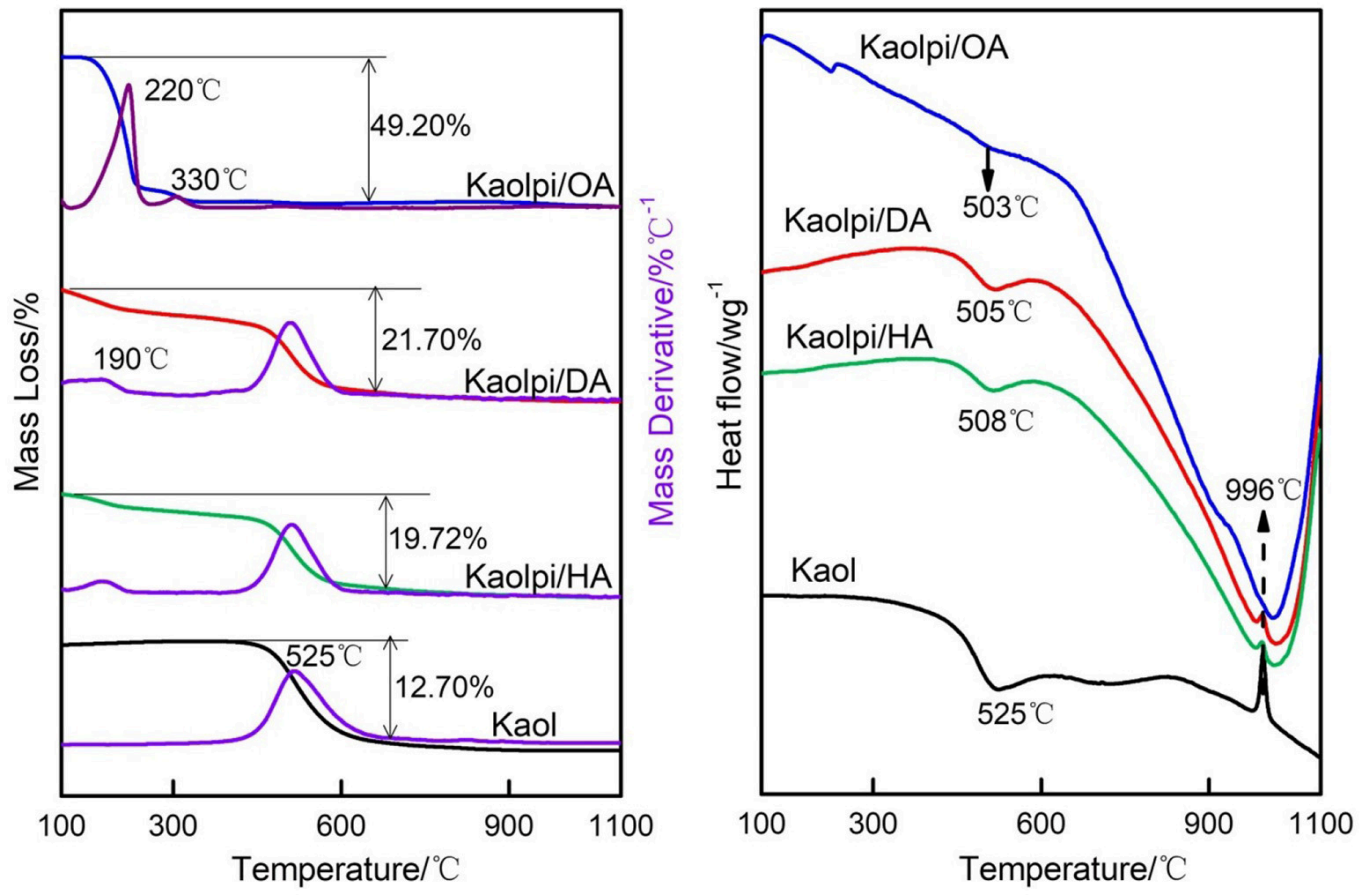
TG-DSC curves for Kaol, Kaolpi/HA, Kaolpi/DA, Kaolpi/OA.

The TG-DSC analysis results of Kaol and its intercalation compounds are shown in Figure 4. The main mass loss of Kaol is the one between 400 and 600°C with the maximum loss rate at 525°C attributed to the loss of water because of the dehydroxylation of the crystal lattice, resulting in the formation of meta-kaolinite (Toussaint et al., 1963; Criado et al., 1984; Sperinck et al., 2011). This loss corresponds to about 12.7% of the total mass which is extremely close to the theoretical reference (13.9%). By comparing the TG-DSC curves of the original Kaol, two main mass loss steps are observed in that of Kaolpi/HA, Kaolpi/DA, and Kaolpi/OA intercalation compounds. The first one occurred between 150 and 350°C, which is due to the decomposition of the intercalated alkylamine molecules (Yuan et al., 2013). The second one occurred at about 500°C is due to the dehydroxylation of the de-intercalated Kaol. For Kaolpi/OA, some OA were intercalated into the silicate interlayer space to form a stable intercalation, which can be proved by XRD pattern. Some molecules are entangled within each other due to the long chain or a clamp in the nanoscrolls that cannot be fully removed (Li et al., 2015; Liu et al., 2016). At 220°C, the distinct mass loss is due to the decomposition of the coated or unbonded OA, while the mass loss at 330°C is attributed to the decomposition of intercalated OA. The exothermic peak at 996°C is result from the recrystallization of Kaol.

By comparing the TG-DSC curves of the original Kaol and Kaol/alkylamine intercalation compounds (Kaolpi/HA, Kaolpi/DA, Kaolpi/OA), one can observe: (a) it has different mass loss steps, as well as a significant difference in the mass losses between Kaolpi/OA and the other two intercalation compounds (Kaolpi/HA, Kaolpi/DA). The mass loss for the decomposition of Kaolpi/HA and Kaolpi/DA were about 20%, but the amount of the two mass losses for Kaolpi/OA is about 50%. This could be owing to a larger amount of OA was intercalated into the silicate interlayer space than that HA and DA (Wang et al., 2017). (b) Dehydroxylation temperature of the intercalation compounds was lower than that of the original Kaol by about 20°C. This is because the intercalation molecules expanded the interlayer spacing, weakened the hydrogen bonds between Kaol layers, bringing about a more facile dehydroxylation from Kaol surface. Therefore, the crystallinity of Kaol sharply decreased after intercalation, which can be confirmed by XRD and IR (below 800 cm^−1^). This result is consistent with the conclusion of some other reports that the temperature of dehydroxylation was determined by the crystallinity of Kaol (Yeskis et al., 1985; Gabor et al., 1995; Sahnoune et al., 2012).

### Decomposition reaction kinetics

The degradation kinetics of the Kaol/alkylamine intercalation compound were investigated by thermogravimetry techniques. The kinetic parameters (activation energy *E* and the pre-exponential factor *A*) of degradation process were calculated on the basic of KAS and Ozawa methods (Kissinger, 1957; Škvára and Šesták, 1975). The thermal degradation mechanism of the Kaol/alkylamine intercalation compound was studied with Satava integral method (Xie et al., 2001; Gao et al., 2005; Zhang et al., 2015).

According to the reaction theory, the non-isothermal decomposition kinetic formula is usually expressed as follows:

(1) $$\begin{array}{r} \frac{\text{d}\alpha }{\text{d}t}=\text{k}\cdot f\left(\alpha \right) \end{array}$$

where α is the extent of conversion rate of B(s) at time *t, f* (α) is the reaction mechanism function, and k is the reaction rate constant. k obeys the following equation:

(2) $$\begin{array}{r} \text{k}=A\text{exp}\left(-\frac{E}{RT}\right) \end{array}$$

where *A, E, R, T* are pre-exponential factor (s^−1^), apparent activation energy (J mol^−1^), universal gas constant (8.314 J mol^−1^ K^−1^), and the temperature of the mass loss (K). Combining Equations (1) and (2) gives the following equation:

(3) $$\begin{array}{r} \frac{\text{d}\alpha }{\text{d}t}=A\exp\left(-\frac{E}{RT}\right)f\left(\alpha \right) \end{array}$$

If the temperature of the sample is controlled at a constant heating rate (β = d*T*/d*t*), the reaction rate can be defined as follows:

(4) $$\begin{array}{r} \frac{\text{d}\alpha }{\text{d}t}=\frac{A}{\beta }\exp\left(-\frac{E}{RT}\right)f\left(\alpha \right) \end{array}$$

After separating the variable, rearranging with integral or differential functions of Equation (4), KAS Equation (5), and Ozawa Equation (6) can be computed as follows:

(5) $$\begin{array}{r} \text{ln}\frac{\beta }{T^{2}}=\text{ln }\frac{AR}{G\left(\alpha \right)E}\;-\;\frac{E}{RT} \end{array}$$

(6) $$\begin{array}{r} \text{ln}\beta =\text{ln }\frac{0.00484AE}{G\left(\alpha \right)R}\;-\;\frac{1.0516E}{RT} \end{array}$$

Where *G(*α*)* represents the integral function of conversion. Because of integral approximation, iterative methods were used to calculate *E* in order to avoid certain deviation. The functions of iterative methods are as follows:

(7) $$\begin{array}{r} \text{ln }\frac{\beta }{{h\left(x\right)T}^{2}}\;=\text{ln }\frac{AR}{G\left(\alpha \right)E}\;-\;\frac{E}{RT} \end{array}$$

(8) $$\begin{array}{r} \text{ln }\frac{\beta }{H\left(x\right)}\;=\text{ln }\frac{0.00484AE}{G\left(\alpha \right)R}\;-\;\frac{1.0516E}{RT} \end{array}$$

Set *x* = *E/RT*, the definition of *h(x)* and *H(x)* are:

(9) $$\begin{array}{r} h\left(x\right)=\frac{x^{4}+18x^{3}+88x^{2}+96x}{x^{4}+20x^{3}+120x^{2}+240x+120} \end{array}$$

(10) $$\begin{array}{r} H\left(x\right)=\frac{\exp\left(-x\right)h\left(x\right)/x^{2}}{0.00484exp\left(-1.0516x\right)} \end{array}$$

The iteration are as the following three steps:

(a) Set *h(x)* = 1 or *H(x)* = 1, calculate *E*_0_ by least squares method according to the slope of the linear relationship lnβ/*T*^2^ and lnβ to 1/*T*.
(b) *E*_0_ and *T* at different α and β were substituted into *x* = *E/RT* and then *x* was substituted into *h(x)* and *H(x)*, the values were substituted into (7) and (8), calculate *E*_1_ by least squares method according to the slope of the linear relationship ln[β*/h(x)T*^2^] and ln[β*/H(x)*] to 1/*T*.
(c) Replace *E*_0_ with *E*_1_, repeat step (b) until *E*_*i*_-*E*_*i*−1_ < 0. 1 kJ mol^−1^. *E*_*i*_ is the exact value of the activation energy of the decomposition reaction.

The *E*-values of Kaol/alkylamine intercalation compounds were calculated by KAS, Ozawa, and iterative methods (Table 2). The calculated results obtained by KAS method were closer to the results from iterative methods, however, the results from Ozawa iterative method showed larger deviation to others. Therefore, the average *E* from iterative methods can be regarded as the activation energy of the decomposition reaction since they are an improvement over KAS and Ozawa methods. The average *E* of Kaolpi/HA, Kaolpi/DA, and Kaolpi/OA are 104.44, 130.80, and 154.59 kJ mol^−1^. It can be seen that the average *E* among of Kaol/alkylamine compounds are positive correlation with the alkyl chain length. One of the reason is that the volatility weakens as the alkyl chain grows. Another reason is that the Kaolpi/OA possesses the best well-ordered intercalation structure which is the most difficult to de-intercalate. And these results can be confirmed by XRD patterns.

**Table 2:** *E* of Kaol/alkylamine intercalation compounds with different α calculated by KAS, Ozawa, and iteration methods.

	**α**	***E*****/(KJ**·**mol**^**−1**^**)**			
		**KAS**	**Ozawa**	**ln[*β/h(x)T^2^*]-1/*T***	**ln[*β/H(x)*]-1/*T***
Kaolpi/HA	0.1	74.92	77.76	75.17	75.18
	0.2	77.35	80.19	77.61	77.62
	0.3	85.44	88.00	85.69	85.69
	0.4	100.23	102.16	100.45	100.45
	0.5	113.52	114.89	113.72	113.72
	0.6	116.15	117.49	116.35	116.35
	0.7	122.54	123.66	122.74	122.74
	0.8	126.32	127.38	126.52	126.52
	0.9	121.48	122.91	121.69	121.70
Kaolpi/DA	0.1	109.36	109.81	109.51	109.51
	0.2	116.52	116.77	116.67	116.67
	0.3	104.95	105.96	105.12	105.13
	0.4	111.42	112.29	111.59	111.59
	0.5	120.44	121.05	120.61	120.61
	0.6	136.10	136.12	136.26	136.26
	0.7	178.68	176.79	178.81	178.80
	0.8	151.39	151.24	151.56	151.56
	0.9	146.88	147.24	147.07	147.06
Kaolpi/OA	0.1	105.52	108.44	105.81	105.81
	0.2	113.12	115.78	113.40	113.40
	0.3	134.68	136.43	134.92	134.92
	0.4	179.42	179.37	179.62	179.63
	0.5	178.76	178.98	178.98	178.99
	0.6	182.19	182.38	182.42	182.42
	0.7	183.42	183.66	183.64	183.64
	0.8	155.85	157.54	156.11	156.11
	0.9	156.15	157.92	156.43	156.43

The functions and *A* of decomposition reaction mechanism was calculated by Satava methods, which was described as follow:

(11) $$\begin{array}{r} \mathrm{lg}G\left(\alpha \right)=lg\;\frac{AE}{\beta R}\;-2.315-\;\frac{0.4567E}{RT} \end{array}$$

Substitute α into every mechanism function *G(*α*)* to obtain the value of lg[*G(*α*)*], linear fitting lg[*G(*α*)*], and 1/*T* which corresponding to the heating rate, the function which has the largest fit can be determined as the most probable mechanism function. And the values of *E* and *A* can be obtained through the slope and intercept of a linear fitting curve.

**Figure 5:**
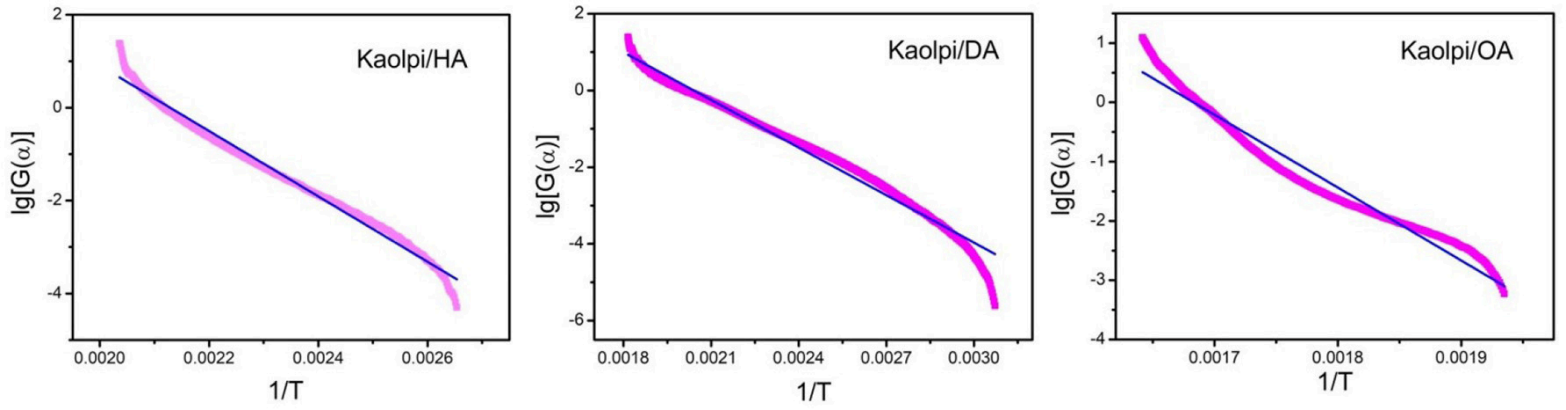
Linear fitting curves of No. 9 function judged by Satava integral method.

As shown in Table 3, five mechanism functions with goodness of fit above 90% are chosen based on Satava integral formula. By comparing there five functions, No. 9 mechanism function *G*(α) = [(1-α) ^−1/3^−1]^2^ with goodness of fit about 97% can be regarded as the most probable mechanism function, and the linear fitting curves of No. 9 function has been shown in Figure 5. *E* and *A* are also in the normal range of decomposition kinetics. The decomposition mechanism functions for the three Kaol/alkylamine intercalation compounds are given in Table 4.

**Table 3:** Selected kinetic mechanism functions judged by Satava integral method.

	**Functions number**	**Integral functions**	**Goodness of fit (%)**	***E* (kJ/mol)**	**Lg*A* (s^−1^)**
Kaolpi/HA	6	${\left[1-{\left(1-\alpha \right)}^{\frac{1}{3}}\right]}^{2}$	93.95	107.15	11.17
	9	${\left[{\left(1-\alpha \right)}^{-\frac{1}{3}}-1\right]}^{2}$	96.50	135.84	15.04
	10	${\left[-\ln\left(1-\alpha \right)\right]}^{\frac{1}{4}}$	95.92	15.02	1.90
	20	[−ln(1−α)]^4^	95.92	240.40	28.32
	28	$\;1-{\left(1-\alpha \right)}^{\frac{1}{4}}\;$	94.54	55.09	5.56
Kaolpi/DA	6	${\left[1-{\left(1-\alpha \right)}^{\frac{1}{3}}\right]}^{2}$	92.18	61.61	5.75
	9	${\left[{\left(1-\alpha \right)}^{-\frac{1}{3}}-1\right]}^{2}$	96.81	77.43	8.06
	10	${\left[-\ln\left(1-\alpha \right)\right]}^{\frac{1}{4}}$	95.03	8.60	1.34
	20	[−ln(1−α)]^4^	95.03	137.62	15.80
	28	$\;1-{\left(1-\alpha \right)}^{\frac{1}{4}}\;$	95.03	31.64	2.89
Kaolpi/OA	6	${\left[1-{\left(1-\alpha \right)}^{\frac{1}{3}}\right]}^{2}$	94.51	179.10	14.96
	9	${\left[{\left(1-\alpha \right)}^{-\frac{1}{3}}-1\right]}^{2}$	96.41	208.90	21.61
	10	${\left[-\ln\left(1-\alpha \right)\right]}^{\frac{1}{4}}$	95.35	26.07	2.31
	20	[−ln(1−α)]^4^	95.35	367.05	38.56
	28	$\;1-{\left(1-\alpha \right)}^{\frac{1}{4}}\;$	94.89	92.90	7.48

**Table 4:** Kinetic mechanism functions of Kaol/alkylamine intercalation compounds.

**Samples**	***E*/kJ mol^−1^**	***A*/s^−1^**	**Kinetic mechanism function**
Kaolpi/HA	104.44	1.09 × 10^15^	$\frac{d\alpha }{dt}=1.64\times 10^{14}\times {\left(1-\alpha \right)}^{\frac{4}{3}}{\left[{\left(1-\alpha \right)}^{-\frac{1}{3}}-1\right]}^{-1}\times \exp\left(-\frac{1.26\times 10^{4}}{T}\right)$
Kaolpi/DA	130.80	1.15 × 10^8^	$\frac{d\alpha }{dt}=1.73\times 10^{7}\times {\left(1-\alpha \right)}^{\frac{4}{3}}{\left[{\left(1-\alpha \right)}^{-\frac{1}{3}}-1\right]}^{-1}\times \exp\left(-\frac{1.57\times 10^{4}}{T}\right)$
Kaolpi/OA	154.59	4.17 × 10^21^	$\frac{d\alpha }{dt}=6.26\times 10^{20}\times {\left(1-\alpha \right)}^{\frac{4}{3}}{\left[{\left(1-\alpha \right)}^{-\frac{1}{3}}-1\right]}^{-1}\times \exp\left(-\frac{1.86\times 10^{4}}{T}\right)$

## Conclusions

The possible structure models and decomposition kinetics for Kaol/alkylamine intercalation compounds have been studied using XRD, FT-IR, and TG-DSC. The intercalation of Kaol/MeOH compound with HA, DA, and OA expands the interlayer spacing of Kaol along the *c*-axis, resulting in the dominant reflection appearing at 2.86, 4.08, and 5.66 nm, respectively. Moreover, the basal spacing after intercalation is not sufficient to allow the alky chain entering by vertical single and bilayer manner. It was concluded that the alky chains of HA, DA, and OA are tilted to the Kaol surface in bilayer and the inclination angle of the alky chains are ~40°.

Based on the KAS, Ozawa, and iterative methods, the results of activation energy *E* of Kaol/alkylamine intercalation compounds were calculated. The average activation energy *E* of Kaolpi/HA, Kaolpi/DA, and Kaolpi/OA are 104.44, 130.80, and 154.59 kJ mol^−1^. The average activation energy *E* among Kaol/alkylamine compounds have a positive correlation with the alkyl chain length. The optimized mechanism function of Kaol/alkylamine decomposition process was determined as a 3D diffusion with the integral function *G(*α*)* = [(1-α) ^−1/3^−1]^2^. This research is advantageous to better understand the decomposition mechanism of clay/organic nanocomposites in addition to provide an inspiration to synthesize new clay-based materials.

## Author contributions

HC and QfL designed the experiment and revised the paper. YZ, QhL, and PX did the experiments and wrote the paper. YZ and QhL contributed equally to this work.

### Conflict of interest statement

The authors declare that the research was conducted in the absence of any commercial or financial relationships that could be construed as a potential conflict of interest.
